# Biological and Exploitable Crossroads for the Immune Response in Cancer and COVID-19

**DOI:** 10.3390/biomedicines10102628

**Published:** 2022-10-19

**Authors:** Letizia Vitali, Alessandra Merlini, Federica Galvagno, Alessia Proment, Dario Sangiolo

**Affiliations:** 1 Candiolo Cancer Institute, FPO-IRCCS, Strada Provinciale 142 Km 3.95, 10060 Candiolo, Italy; 2 Department of Oncology, University of Turin, Regione Gonzole 10, 10043 Orbassano, Italy

**Keywords:** COVID-19, SARS-CoV-2, cancer, immunity, immunotherapy

## Abstract

The outbreak of novel coronavirus disease 2019 (COVID-19) has exacted a disproportionate toll on cancer patients. The effects of anticancer treatments and cancer patients’ characteristics shared significant responsibilities for this dismal outcome; however, the underlying immunopathological mechanisms are far from being completely understood. Indeed, despite their different etiologies, SARS-CoV-2 infection and cancer unexpectedly share relevant immunobiological connections. In the pathogenesis and natural history of both conditions, there emerges the centrality of the immune response, orchestrating the timed appearance, functional and dysfunctional roles of multiple effectors in acute and chronic phases. A significant number (more than 600) of observational and interventional studies have explored the interconnections between COVID-19 and cancer, focusing on aspects as diverse as psychological implications and prognostic factors, with more than 4000 manuscripts published so far. In this review, we reported and discussed the dynamic behavior of the main cytokines and immune system signaling pathways involved in acute vs. early, and chronic vs. advanced stages of SARS-CoV-2 infection and cancer. We highlighted the biological similarities and active connections within these dynamic disease scenarios, exploring and speculating on possible therapeutic crossroads from one setting to the other.

## 1. Introduction

The outbreak of novel coronavirus disease 2019 (COVID-19) caused by severe acute respiratory syndrome coronavirus 2 (SARS-CoV-2) has been categorized as a pandemic since March 2020. After two years, it has resulted in more than 300 million infections and 5.5 million deaths worldwide. Cancer patients have been particularly affected by the pandemic, both directly, due to immunosuppression caused either by the neoplastic disease or by the cancer treatment itself, and indirectly, with delays in diagnosis and postponed follow-ups, difficulties in organizing and conducting clinical trials and the need to implement remote telemedicine approaches in such a frail population [1,2,3,4,5]. At the molecular level, SARS-CoV-2 infection involves the spike protein (S), which recognizes and binds to the cell surface receptor angiotensin-converting enzyme 2 (ACE2) [6,7], allowing the virus to enter the host cell. After initial replication of the virus in the upper respiratory tract, viral replication can spread to the lower respiratory tract [8]. COVID-19 patients might experience either asymptomatic or mild forms of the disease, or severe disease manifestations requiring hospitalization and mechanical ventilation. Some severe COVID-19 patients display acute respiratory distress syndrome (ARDS), which reflects severe respiratory damage [9]. It has been observed that patients with co-morbidities are more susceptible to severe symptoms that may ultimately lead to multiple organ dysfunction and poor prognosis [10]. Indeed, cancer patients represent a significant proportion of patients in vulnerable/fragile groups. Data reporting a precise association between an increased infection incidence and the oncological disease are still inconclusive, since it is rather challenging to draw conclusions in this complex (and heterogeneous) clinical context. Nevertheless, several studies report that cancer patients have a significantly higher mortality rate and adverse outcomes from COVID-19, in particular those patients who are receiving or have just received anticancer treatment [5,11].

Indeed, the factors which can affect COVID-19 infection outcome and survival in cancer patients can be divided according to intrinsic factors derived from patients’ characteristics and comorbidities, and “extrinsic” factors related to the type of anticancer treatment and concomitant medications that the patient is receiving or has just received at the time of COVID-19 infection. Concerning the first group (“intrinsic factors”), it has been shown that both diabetic and obese patients (often concomitantly affected by the metabolic syndrome) are at increased risk of severe COVID-19 infection and COVID-19 related death [12,13]. However, the thorough ESMO-CoCARE study on COVID-19 in cancer patients [14] has reported better outcomes from COVID-19 (lower hospitalization rate) for overweight and obese patients [14]. The so-called “obesity paradox” in cancer has long been debated, with initial evidence hinting at favorable outcomes for overweight and obese patients, possibly due to improved tolerance to anticancer treatments [15]. A large meta-analysis from 203 studies has concluded that in lung cancer, melanoma, and renal cell cancer obesity showed a protective effect in terms of outcome, while in most other cancer types, obesity was associated with reduced survival and increased relapse risk [16]. Elderly patients, a fragile category per se and especially in the context of COVID-19 infection [17], are particularly hit by the cardiovascular effects of COVID-19 [18], and their risk of contracting severe COVID-19 is also increased by immunosenescence-related factors [19]. Another independent clinical condition which has been associated with poor outcomes in SARS-CoV-2 infection is “Low T3 Syndrome”, possibly due to the interplay between thyroid dysfunction and the immune system functioning correctly in acute infection [20].

The “extrinsic” variables influencing the outcome of cancer patients with COVID-19 infection encompass treatment-related conditions, ranging from the cardiotoxic [21,22] to immunosuppressive and immunomodulatory effects of cancer therapeutics and concomitant medications such as corticosteroids [23,24,25,26,27]. Given the well-known acute and long-term cardiovascular complications from COVID-19 [28,29,30], any condition contributing to worse cardiovascular outcomes (such as previous or concurrent cardiotoxic treatment) must be carefully considered. The main classes of cardiotoxic oncological treatments include anthracyclines [31] and anti-HER2 agents [32], while all cytotoxics have well-recognized immunosuppressive effects in cancer patients [27]. What is more, biological treatments directed against B cells, such as rituximab, an anti-CD20 monoclonal antibody, can put patients (even after vaccination) at an increased risk of severe COVID-19 and death [33,34]. These data have prompted consideration on how to best protect cancer patients from severe/fatal sequelae of COVID-19 infection, while ensuring the delivery of effective, essential anticancer treatments [35]. 

Although COVID-19 and cancer obviously have different etiologies, they both share some key biological and immunological mechanisms, especially in their advanced stages. In particular, immune dysregulation plays a crucial role in both diseases in their advanced forms, leading to processes of chronicization and disease progression. Among multiple factors and elements that are involved in the functions and dysregulation of the immune system, cytokines and immune checkpoints are crucial pillars that play a relevant role in both conditions. For instance, the typical features of immune dysfunction shared by SARS-CoV-2 infection and cancer are the deregulated production of cytokines, along with type I IFN-mediated immune responses (essential to tackle both COVID-19 and cancer), and the immune exhaustion that may occur in these conditions [36]. 

The aim of this work is to highlight and review the main immunobiological connections between COVID-19 and cancer. We will underline the considerations on the “time variable” that orchestrate the appearance, functional and dysfunctional roles of multiple immune effectors of acute and chronic responses potentially implicated in both COVID-19 and tumoral settings. Within this frame, the main cytokines and molecular pathways involved in the pathogenesis and clinical features of both conditions will be discussed. We will conclude by reviewing the initial evidence and speculating on potential therapeutic exploitations of the emerging immune biological intersections between COVID-19 and cancer. 

## 2. Time Makes the Difference: Acute and Chronic Features of Immune Response

The acute immune response is part of a complex biological process triggered by cellular damage, caused either by sterile injury (cell death) or infection. After cellular damage, the immune system attempts to eliminate or neutralize injurious stimuli and initiates recovery and regenerative processes [37]. 

Immediately upon injury, factors released from damaged epithelial and endothelial cells, along with cytokines and chemokines promote the migration of neutrophils and monocytes to the site of inflammation. The cytokines that are best known for stimulating and perpetuating inflammatory responses are interleukin, (IL)-1, IL-2, tumor necrosis factor (TNF)-α, IL-6, type I and II interferons (IFN-I and IFN-II), and transforming growth factor (TGF)-β [38]. The first cells attracted in loco are neutrophils, then monocytes, natural killer (NK) and mast cells [39]. During the early phase of an infection, the efficacy of the innate immune response, mainly mediated by IFN-I, plays an essential role in preventing viral replication, T cell exhaustion, and cytokine overproduction [40,41]. Moreover, it is crucial to support the subsequent adaptive immune response and eventually the clinical outcome [42,43]. It has been proved that a performing and coordinated cellular immune response is crucial to control the disease and eliminate it, in this case, SARS-CoV-2 infection [44]. The adaptive immune system is mainly constituted by three cell types: B cells which produce antibodies, CD4+ T cells which exert multiple helper and effector features and CD8+ T which kill infected and target cells through perforin and granzymes secretion [45,46]. Virus-specific cells derive from selected and expanded pools of naïve B and T cells which can target precise molecular structures. The extensive proliferation and differentiation of specific effector T cells (including helper T cells and cytotoxic T cells) and effector B cells (plasma cells) is a time-consuming process (∼6–10 days after priming) which, once completed, provides viral clearance [47,48,49].

**Figure 1:**
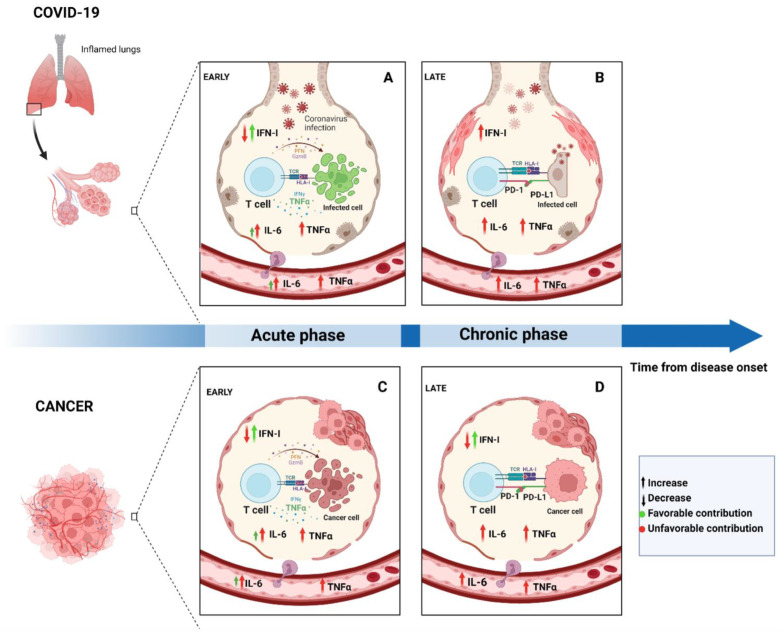
Time makes the difference. Schematic representation of the acute and chronic phases of inflammation in COVID-19 (**A**,**B**) and cancer (**C**,**D**), in the alveolar and tumoral microenvironments, respectively. Created with Biorender.com.

In COVID-19, SARS-CoV-2 specific antibodies, CD4+ and CD8+ T cells are crucial for infection resolution [50]. Remarkably, several studies report that specific T cell responses correlate significantly with milder disease [44,51,52,53,54] and effective viral clearance [55]. Notably, lower COVID-19 disease severity has been better associated with SARS-CoV-2 specific CD4+ T cells than CD8+ T cells and antibodies [44], whose main cytokine produced is IFN-γ [44,50,56]. Timing is again crucial; indeed a study demonstrated that a fast induction of specific CD4+ T cells during the acute phase of the disease correlates well with milder COVID-19 and prompt viral clearance [57]. CD8+ T cell-mediated immune response develops rapidly in acute COVID-19, exerting relevant cytotoxic effects through IFN-γ, perforin and granzyme B [44,51,58]. It has also been reported that CD8+ T cells decreased in people who experienced SARS-CoV-2 infection; on the other hand, in healthy vaccinated subjects, CD8+ T cell expression increased significantly [59]. Another important T cell population is that of the regulatory T cells (Tregs), whose role during COVID-19 is still debated. A study described a significant reduction of Tregs in COVID-19 severe cases [60]. Lower levels of Tregs may be the cause of the overactivation of the immune system and subsequent lung injury in COVID-19 patients [61]. On the other hand, a study reported that Treg levels remarkably increased in moderate and severe SARS-CoV-2 infection, along with their IL-10 production [62]. Moreover, highly activated Tregs with an enhanced expression of CD25 have been described in severe COVID-19 patients [63]. Overall, the deficit in the development of the adaptive immune response may exacerbate the predominant inflammatory immune response which can cause substantial tissue damage. Thus, negative regulatory mechanisms are crucial during innate and adaptive immune responses to minimize detrimental effects and optimize the host defense [48]. In fact, the perfect balance between innate and adaptive immunity can be reached when they synergize with negative control and immunosuppressive mechanisms. Immune checkpoints are an emblematic example; indeed, during chronic infections, they hamper the excessive antiviral response in order to prevent immunopathological damage [64]. Alterations during the inflammatory process may lead to chronicization and may be associated with chronic inflammatory diseases, cancer, autoimmune and degenerative diseases [39]. In Figure 1, the progressive chronicization of the inflammatory process is depicted for both COVID-19 and cancer scenarios. 

## 3. Cytokines

During the early stages of the pandemic, increased levels of many cytokines, including IL-6, IL-1β, TNF-α, and interferons, were observed in patients with COVID-19 [65,66]. These molecules can potentially serve—in general—as disease biomarkers for diagnosis, prognosis, and therapeutic decision-making. Elevated cytokine levels may cause a systemic syndrome, known as a cytokine release syndrome (CRS). CRS results from an excessive and dysregulated immune response, and it is thought to cause a significant increase in proinflammatory cytokines triggered by many factors such as infections and certain drugs [67,68]. It has been reported that levels of proinflammatory cytokines (IL-2, IL-6, IL-7, IFN-I, IFN-II) are strictly correlated with the viral load and lung injury in patients with severe COVID-19, reflecting the severity and the prognosis of this disease [69,70,71]. Chronic inflammation, of which CRS may be an expression, could act as a biological link with cancer, contributing to the neoplastic change. It is possible to include several examples, such as pancreatitis in pancreatic cancer, AIDS in Kaposi’s sarcoma, tobacco in lung cancer, viral hepatitis in liver cancer [36,67]. To understand how to interrupt this process, it is key to understand which factor(s) drive this transition and how this might be inhibited. In this review we will focus on the main players, such as IL6, TNF-α, and IFNs, which deserve a dedicated analysis of their specific roles in chronic inflammation and immunopathological reactions. 

**IL-6** is a pleiotropic cytokine with nearly ubiquitous expression in stromal and immune cells. It transmits defense signals from a pathogen invasion or tissue damage site to stimulate acute phase reactions, immune responses, hematopoiesis, and various internal organs to prepare for host defense [72]. However, excessive and sustained production of IL-6 is associated with various inflammatory diseases [73]. IL-6 is involved in the proliferation and differentiation of malignant cells and found to be high in the serum and tumor tissues in the majority of cancer patients, including colorectal cancer, breast cancer, prostate cancer, ovarian carcinoma, pancreatic cancer, lung cancer, renal cell carcinoma, cervical cancer and multiple myeloma. Elevated or lower levels of circulating IL-6 may be, respectively, negative or positive prognostic indicators for survival and predictors of response to therapy [74,75]. 

Regarding COVID-19, in a meta-analysis review, it has been demonstrated that serum levels of IL-6 are significantly increased in severe forms of COVID-19. Those levels are indeed associated with adverse clinical outcomes, including intensive care unit (ICU) admission, ARDS and death. In fact, IL-6 serum levels in patients affected by severe COVID-19 are about three times higher with respect to IL-6 levels of patients with mild disease presentation. Another study suggests that elevated IL-6 levels could be considered as an independent risk factor for disease severity and in-hospital mortality, and that dynamic IL-6 changes may serve as a potential predictor for lung injury [76]. 

**TNF-α** is a pivotal pro-inflammatory cytokine. It is released by various cell types (e.g., monocytes, macrophages), and promotes inflammation by further inducing the release of pro-inflammatory cytokines, prompting the activation and proliferation of naïve and effector T cells, but also inducing apoptosis of highly activated effector T cells, further determining the size of the pathogenic or protective conventional T cell pool [38,77]. Alongside other cytokines, TNF-α is involved in the regulation of inflammatory processes, infectious diseases, and malignant tumors [78]. TNF is able to act as an endogenous tumor promoter to bridge inflammation and carcinogenesis. The role of TNF in tumorigenesis, tumor growth and metastasis has been demonstrated in vitro and in various mouse tumor models, including colon cancer [79] and skin cancer [80]. In several studies, it has also been reported that serum TNF concentration is increased in different cancer patients; moreover, its concentration was markedly decreased during chemotherapy in breast [81] and prostate cancer patients [82], correlating well with response to treatment, suggesting that serum TNF levels could be a potential predictive biomarker in these cancer types. High levels of TNF expression in tumor tissues are associated with malignancy progression, too, and have been reported in chronic lymphocytic leukemia [83], prostate cancer [82], and other cancer types [84]. Concerning the TNF role in COVID-19, a recent meta-analysis has highlighted a peculiar pro-inflammatory cytokine profile in patients with severe forms of the disease, and TNF-α was recognized as one of the prevailing cytokines during the COVID-19 cytokine storm [85]. Indeed, one significant mechanism with which TNF-α mediates lung inflammation and ARDS, appears to be the reduced CD4^+^ and CD8^+^ T-cell counts in patients with severe COVID-19. More particularly, CD4^+^ T-cells lead immune responses against viral infections and CD8^+^ T-cells play a role of essential importance in the host’s defense against respiratory viruses, providing viral clearance and participating in the containment of secondary infections. Furthermore, its concentration was found to increase in both the early and late stages of SARS-CoV-2 infection [85].

**IFNs** belong to three different families, type I, II and III IFNs, according to their receptor specificity and sequence homology. Type I IFNs are a multi-gene cytokine family comprising a single IFNβ gene and 13 partially homologous IFNα subtypes in humans. They signal through a common receptor, IFNR, which is formed by the heterodimerization of IFNAR1 and IFNAR2 [86]. The type II IFN family includes a single gene product, IFNγ, mainly produced by T cells and NK cells, which exerts its effect on cells expressing IFNγ receptor (IFNγR) [87]. Type III IFNs comprise IFNλ1, IFNλ2 and IFNλ3, with comparable functions to type I IFNs. Since the expression of their receptor is limited to epithelial cells, their effects are restricted [88]. In this work, we mostly focus on type I IFNs (hereafter referred to as IFN-I), because of their central role in cancer and in COVID-19. After IFN-I binding to IFNAR1/2, IFN-I signaling leads to the activation of a wide range of interferon regulatory factors (IRFs) and IFN-stimulated genes (ISGs), promoting inflammatory and innate antiviral responses [89]. Immune responses against both cancer and infectious diseases are strictly dependent on IFN-I effects [87,90,91]. In cancer biology, IFN-I has a crucial role in inhibiting tumor proliferation, promoting tumor cell senescence and death, and controlling cancer stem cell growth. Moreover, impaired IFN-I signaling is associated with tumor progression [92,93]. It has also been reported that the efficacy of various therapeutics against cancer, comprising cytotoxic drugs, radiotherapy, targeted therapy and immunotherapy depend on functional IFN-I signaling, to enhance tumor cell inhibition and effective antitumor immune response [94]. The vital and positive role of IFN-I responses during the early phase of viral infection has been widely demonstrated. Some studies support the hypothesis that SARS-CoV-2, as other viruses, has developed mechanisms to escape antiviral responses in the host. In fact, a study reported that IFN-I signaling was hampered during COVID-19 infection [42]. More specifically, several studies reported a different IFN-I dysregulation according to the severity and the stages of the disease; during the early phases, mild and moderate COVID-19 patients showed higher IFN-I levels in peripheral blood and in the site of infection. On the other hand, IFN-I expression seemed to be suppressed in severe cases of COVID-19 patients, mainly older people with comorbidities, who presented with higher viral load [45]. This suggests that the timing and the role of IFN-I response is crucial during infection: IFN-I is decisive in the early stages to promote an inflammatory response; in fact, reduced IFN-I levels in infected patients may be a warning signal of disease severity; by contrast, an excessive response in late stages would aggravate the inflammation and the progression of COVID-19 and worsen the physiopathology [43,95,96]. This information found support in mouse models too; a delayed IFN-I response could not indeed achieve the inhibition of viral replication of SARS-CoV-2 [97].

## 4. Molecular Pathways

Infections and cellular damage activate acute immune response, during which, as we have seen, cytokines and chemokines promote recruitment of leukocytes to the site of infection or injury, pursuing the inflammatory responses. Primary inflammatory stimuli, including microbial products and cytokines such as IL-1β, IL-6, TNF-α, mediate inflammation through specific receptors, among which toll like receptors (TLR), IL-1 receptor (IL-1R), IL-6 receptor (IL-6R), and the TNF receptor (TNFR) are amongst the most important ones [38]. Receptor activation triggers important intracellular signaling pathways, including nuclear factor kappa-B (NF-κB), and Janus kinase (JAK)-signal transducer and activator of transcription (STAT) pathways, which are the most relevant. Those inflammatory pathways exert their signaling function through transcription factors which regulate a variety of inflammatory genes, including cytokine ones such as IL-1, TNF-α, IL-6, and IFNs, that may be finally released at the site of inflammation [75,98]. Dysregulation of NF-κB, or JAK-STAT activity is associated with inflammatory, autoimmune, and metabolic diseases, and cancer [99]. Likewise, as it has been done for cytokines, in this section we will discuss the interplay of COVID-19 and cancer at the intracellular level, analyzing some of most important inflammatory pathways. 

**NF-κB** transcription factor plays important roles in inflammatory, immune response, survival, cell growth and apoptosis processes and can be activated by viral genetic material or proteins [100]. The NF-κB family includes five related transcription factors: RelA (p65), RelB, NF-κB1 (p50 and its precursor p105), NF-κB2 (p52 and its precursor p100), and c-Rel homo/heterodimers with RelA or RelB [101]. NF-κB signaling requires IKK subunits, which regulate pathway activation through IκB phosphorylation. This pathway is triggered by TLRs and inflammatory cytokines; it dictates, in turn, the expression of cytokines, as a positive feedback mechanism, and inflammatory cell recruitment, contributing to the inflammatory response. NF-κB is a central mediator of pro-inflammatory gene induction and functions in both innate and adaptive immune cells [98]. It has been reported that NF-κB pathway is activated in SARS-CoV-2 infected cells [102]. Moreover, a recent study demonstrated that SARS-CoV-2 accessory nucleocapsid proteins are able to activate NF-κB function and increase proinflammatory cytokine expression [103]. Apart from its association with the “cytokine storm”, the NF-κB pathway is associated with the pathogenesis of the metabolic syndrome, including diabetes and obesity, which may cause in turn atherosclerosis and vascular endothelial damage. These metabolic conditions initiate and propagate hyperactivation of the NF-κB pathway, contributing to worse outcomes and the severe disease manifestations observed in obese and diabetic patients with COVID-19 [104,105,106]. Dysregulation of this pathway has been reported in many chronic diseases, including cancer. Members of the NF-κB protein family are in fact mutated in several types of malignancies, especially hematopoietic ones. For example, the retroviral oncogene v-Rel and the mutated form of its homolog c-Rel can induce lymphoid tumors [107]. Rearrangements and amplifications of the latter are frequent in non-Hodgkin’s B-cell lymphomas [108], while NF-κB2 is often activated through chromosomal translocation in lymphomas and leukemias as well [109]. Concerning solid tumors, the deletion of the IκBα gene has been reported in patients with glioblastoma, and IKK1, IKK2 and IKK mutations have been observed in breast and prostate cancer [110,111,112,113]. However, the constitutive NF-κB activation in solid tumors may be influenced by the proinflammatory tumor microenvironment rather than genetic mutations. The infiltration of immune cells secreting pro-inflammatory cytokines contribute to NF-κB activation, generating a pro-tumorigenic microenvironment, in which the immune system may be disabled and genomic instability and genetic mutations may occur, promoting both tumor initiation and tumor progression [114,115,116].

**JAK-STAT**- The JAK-STAT signaling pathway is extremely conserved and represents a focal node of cell function. It involves more than 50 cytokines, such as hormones, interferons such as IFN-I, interleukins such as IL-6, and growth factors [117]. Thanks to this pathway, extracellular factors can control downstream gene expression and consequent events including hematopoiesis, immune competence, tissue repair, apoptosis and inflammation [118,119]. JAKs are associated with cytokine receptors, and often mediate their tyrosine phosphorylation, as in the case of type 1 IFNs receptors (composed of two subunits, IFNAR1 and IFNAR2), and IL-6 receptor IL-6R. Once these receptors are activated by their ligands, they phosphorylate one another, creating binding sites for one or more STAT proteins, which are constitutively inactive cytoplasmic transcription factors. Recruited STATs undergo phosphorylation and dimerization and are then transported into the nucleus to regulate specific genes, transforming an extracellular input into a transcriptional response [120]. The JAK-STAT signaling pathway is activated in hematological malignancies and, in particular, the somatic JAK2V617F mutation can be found in >95% of patients affected by polycythemia vera [121,122,123]. Activating STAT mutations, even if they are rare, have been reported in large granular lymphocytic leukemia, specifically in STAT3 in 40% of patients [124]. STAT protein activation appears frequently in solid tumors, resulting in different clinical implications. For example, the activation of STAT3 or STAT5 is associated with worse outcomes in non-small cell lung cancer [125] prostate cancer [126] oral squamous cell carcinoma [127] and melanoma [128]. By contrast, in breast cancer [129] and in some studies in colorectal cancer [130], those mutations seem to be correlated with better prognosis. Besides the role of JAK-STAT in CRS downstream IL-6 axis activation in COVID-19, it has been reported in a recent study that SARS-CoV-2 may disrupt JAK-STAT pathway components as a counteractive action to tackle IFN-mediated antiviral responses, facilitating virus replication in diverse tissue types [131].

**PD-1/PD-L1 axis**- The PD-1/PD-L1 axis represents the epitome of immune checkpoints in immunobiology. This peculiar signaling pathway had been discovered well before the advent of the COVID-19 pandemic: in the nineties, the role of immune checkpoints in cancer was elucidated thanks to the pivotal intuitions of the Nobel prize winners James P. Allison and Tasuku Honjo [132]. PD-1 is a transmembrane receptor expressed by activated T cells, whose known ligands (PD-L1 and PD-L2) are expressed on both tumor and stromal cells. Its main action is to mediate immunosuppression; by being an “immune checkpoint” at the end of the immune activation cascade, it provides an ultimate signal to innate immune cells and eventually falls under the radar of T cells, enabling cancer cells to evade both innate and adaptive immunosurveillance [133]. For what concerns the role of PD-1/PD-L1 in COVID-19, it should be noted that one of the first clinical and laboratory observations in patients infected by COVID-19 was the finding of lymphopenia, and the correlation of worsening lymphopenia with disease progression and disease severity [65,134]. Indeed, in COVID-19 patients, and especially in those requiring ICU, lymphopenia has been associated with T-cell exhaustion which is mediated, among others, by the PD-1/PD-L1 axis. Cytofluorimetric analysis of samples derived from COVID-19 patients with severe infection and hospitalized in ICU, with respect to healthy controls and patients with less severe disease manifestations, has shown significantly higher PD-1 expression levels on CD8+ and CD4+ cells from ICU patients [135]. Blood samples analysis from COVID-19 patients vs. healthy control subjects has also shown soluble PD-L1 serum upregulation in SARS-CoV-2 infected patients, as determined by enzyme-linked immunosorbent assay (ELISA) [136]. 

**Poly-ADP-Ribose Polymerase (PARP) enzymes**- This unique protein family of enzymes is in charge of a peculiar form of post-translational modification, PAR(Poly-ADP Ribose)ylation, which consists of the addition of negatively charged, ADP-ribose polymer chains to target proteins involved in processes as diverse as DNA repair, epigenetic modifications, and inflammation [137,138]. Intriguingly, the Mac1 macrodomain of SARS-CoV-2 is a mono-ADP-ribosyl hydrolase, which binds to and hydrolyzes mono-ADP-ribose (MAR) residues on target proteins, with a disruptive action on MARylation as a form of post-translational modification [139]. Indeed, MARylation—similarly to PARylation—is an activity of several PARPs, which are induced also in the acute inflammation phase in response to viral infection through IFN signaling [140]. The PARP family encompasses 17 different enzymes, requiring NAD+ for proper enzymatic activity [141], among which five catalyze PARylation (PARP1, PARP2, PARP5A, PARP5B) while most other members show MARylating activity (PARP3, PARP4, PARP6, PARP14, PARP15) [140,142,143,144]. As far as cancer pathobiology is concerned, the role of PARPs is mainly triggered by supervening DNA damage and repair mechanisms; in cells bearing homologous recombination (HR) defects, as in the case of BRCA1/2 mutations, the role of PARP becomes even more important in supporting alternative DNA repair pathways. Hence, PARPs have become attractive targets in cancer therapeutics for the possibility of inducing the so-called synthetic lethality [145] in cells bearing HR defects, by inhibiting PARP activity and thus leading to cell death upon accumulation of unrepaired double strand breaks.

## 5. Therapeutic Implications

Cytokines and cytokine pathways have been thoroughly investigated both as therapeutic allies and as therapeutic targets in oncology and oncohematology in the last four decades. However, many of these treatments failed to be implemented in clinical practice either due to low efficacy, or significant side effects [146]. Obviously, many drugs targeting cytokines have been developed for autoimmune diseases, rather than cancer treatment, with the advent of the so-called “biological drugs” in rheumatology. A few of these molecules have been translated to COVID-19 treatment, as for instance anti IL-6 monoclonal antibodies, anti-JAK1/2 small molecule inhibitors, both approved by national and international drug regulatory agencies for treating autoimmune conditions such as rheumatoid arthritis [147,148]. In COVID-19, timing is crucial with respect to the use of anticytokine treatments; indeed, immunity system priming is necessary at the early stages of the infection during the viral shedding; consequently, cytokine-based therapies may be eligible to promote inflammation. However, hyperinflammation and CRS must be avoided given the potential side effects in the severity of COVID-19, and so blocking cytokine receptors or their pathway may be a suitable choice.

**Type I IFNs**—In order to trigger the innate immune response, recombinant IFN-Is, such as IFNα and IFNβ, have been widely used in the past and studied in cancer treatment alone or in combination with chemotherapeutics, both in oncology and oncohematology [149,150,151,152,153,154]. Recombinant IFN-Is are also being actively studied as therapeutic approaches for COVID-19; in particular, a few studies have assessed the effectiveness of nebulized IFNβ-1a in COVID-19 hospitalized patients. These studies highlight that receiving IFNβ-1a is correlated with earlier recovery and lower incidence of adverse events and has also been associated with clinical improvement in one study [155,156]. Recent studies and a meta-analysis underline the positive effects of IFNβ administration combined with other antivirals, such as ribavirin in the early days of virus spreading, resulting in higher survival rates, lower mechanical ventilation rates, enhanced viral clearance and antiviral response [157,158]. Similarly, a multicenter cohort study investigated the correlation between early administration of IFNα-2b and antivirals (lopinavir/ritonavir) with a lower mortality rate when compared with antiviral treatment alone; conversely, patients with late administration of IFN experienced increased mortality and delayed recovery among survivors [159].

**Targeting IL-6/JAK/STAT**—One of the main players of hyperinflammation and CRS is IL-6; its overproduction was observed in patients with severe COVID-19 and in patients with several types of cancer, as reported above. Therefore, drugs targeting the IL-6/JAK/STAT axis developed in the past to cope with other diseases (mainly autoimmune diseases), are under analysis for COVID-19 treatment. Several IL-6R inhibitors have been developed during the years such as sarilumab, saltuximab and tocilizumab, and during the summer of 2021, they have been recommended for COVID-19 treatment [160]. Tocilizumab has been approved by the FDA for use in adult patients with rheumatoid arthritis, and for the management of cytokine-release syndrome in adult or pediatric patients receiving treatment with CAR T cells in hematological malignancies. Preclinical studies suggest that tocilizumab might be effective against ovarian [161], pancreatic [162], and colitis-associated colorectal cancer arising in an inflammatory bowel disease background [163]. In June 2021, the US Food and Drug Administration issued an emergency use authorization for the use of tocilizumab in combination with corticosteroids in hospitalized adult and pediatric patients with COVID-19 who required non-invasive or invasive mechanical ventilation or extracorporeal membrane oxygenation. In fact, the experience with tocilizumab showed a clinical improvement in a large proportion of patients with severe pneumonia. A meta-analysis of 13 studies indicated that patients treated with tocilizumab had lower mortality when compared with control groups receiving standard COVID-19 treatment or no treatment [164]. What is more, a multicentric study demonstrated that tocilizumab reduced mortality and disease severity in a dose- and time-dependent way. The study revealed that the IL-6R inhibitor gave better results in severely ill patients than moderately ill ones, since they were in an advanced stage of inflammation. In fact, the best timing for tocilizumab administration was after 8 days, when the inflammation becomes prevailing and the immunopathologic damage starts to develop. On the other side, they also highlighted that the limited effect of tocilizumab during earlier stages could be due to the lower inflammatory conditions at the viral shedding, whereas the limited activity during later stages may take place because the inflammation is by then irreversible [165].

Another way of intervention on the IL-6 axis is the intracellular inhibition of JAK 1/2 kinases. The most used inhibitors are ruxolitinib and baricitinib, small molecule tyrosine kinase inhibitors of JAK1 and JAK2. The first one has been adopted for cancer therapy to treat myeloproliferative neoplasms such as myelofibrosis [166,167], while baricitinib was approved for the treatment of rheumatoid arthritis (RA) [168], inflammatory bowel diseases (IBD) and psoriasis [169]. Considering the role of the JAK/STAT pathway in inflammation and CRS, both inhibitors have been widely used and studied in clinical trials for COVID-19 treatment. Despite their similar activity, only baricitinib has been recommended by the WHO, in January 2022 [170]. In a multicenter clinical study, ruxolitinib showed a fast anti-inflammatory effect and a slightly lower mortality rate in severe COVID-19 patients, although it has failed to reduce ICU admissions and mechanical ventilation [171]. These results are also in line with the phase III clinical trial RUXCOVID, which failed in the achievement of its primary endpoint: in March 2022, the final results of RUXCOVID were published, highlighting the absence of benefits of ruxolitinib in addition to standard care in the overall patient population [172]. On the other hand, concerning baricitinib, a phase III clinical trial showed the efficacy and safety of this inhibitor combined with standard of care for the treatment of hospitalized adults with COVID-19 [173]. Moreover, a recent meta-analysis found that baricitinib improved the mortality rate, ICU admission, invasive mechanical ventilation requirement and the oxygenation index [174]. An observational retrospective study confirmed these data in severe COVID-19 cases, highlighting the improvement in recovery time and the absence of relevant adverse events [175].

**TNFα inhibitors**—The most well-known TNFα inhibitors are infliximab and adalimumab, monoclonal antibodies developed in the last decades to cope with chronic inflammatory disorders such as RA, IBD, psoriasis, psoriatic arthritis [176]. Surprisingly, among those chronic conditions of inflammation and cytokine dysregulation, cancer is missing. On the contrary, the use of these TNF inhibitors is questionable; in several studies it has been reported that they may promote lymphoproliferative disorders and other malignancies in RA patients [177,178], although this finding was not validated by all research groups [179]. Regarding COVID-19 treatment, instead, several studies have shown how infliximab and adalimumab may protect patients with chronic inflammatory diseases from severe COVID-19. In fact, a meta-analysis reported that the standard administration of TNFα inhibitors in those patients was associated with lower hospitalization probability and ICU admission [180]. Moreover, a case-control study highlighted that TNFα inhibitors, adalimumab in particular, significantly decreased the risk of developing COVID-19 in patients with RA [147]. As far as TNFα inhibitors exclusively meant for an anti-COVID-19 treatment are concerned, a clinical trial evaluating adalimumab has been reported in China (ChiCTR2000030089). Furthermore, a completed phase II clinical trial showed that infliximab may extinguish the pathological inflammatory signaling providing clinical recovery in severe COVID-19 [181]. Indeed, the results of further clinical trials are necessary, to better elucidate the role and the timing of TNF inhibitors in COVID-19 treatment.

**NF-κB inhibition**—TNFα is one of the proinflammatory cytokines which can trigger the activation of NF-κB pathway, crucial during CRS, whose dysfunction is involved in tumor development. NF-κB-associated inflammation may be minimized through modulation at the level of NF-κB activation itself, IκB degradation and trafficking, along with TNFα inhibition, as mentioned above [182]. It has been reported that the blockage of XPO1 may support NF-κB pathway suppression, preventing the export of IκB from the nucleus. selinexor and verdinexor are selective inhibitors of nuclear export (SINE) that show antitumor activity by preserving the correct localization of tumor-suppressor proteins [183]. Selinexor has exhibited effectiveness in patients with hematological malignancies such as multiple myeloma [184,185] and solid tumors [186]. Besides the antitumoral aspect, SINE compounds have shown antiviral effects in influenza virus models and anti-inflammatory properties against ARDS, the same dismal (and often deadly) complication of SARS-CoV-2 pneumonia [187]. Based on this information, several clinical trials have been registered to evaluate SINE compounds effectiveness and safety in COVID-19 patients (NCT04349098, NCT04349098, NCT04355676). An important NF-κB inhibitor is cromolyn, a synthetic compound used to treat asthma which showed potent anti-inflammatory and antitumoral effects [188]. It has been reported that cromolyn inhibits elevated basal activity of NF-κB pathway in pancreatic tumor cells in vivo and in vitro. Moreover, it has proven effective in reducing inflammation in several diseases such as amyotrophic lateral sclerosis [189], and Alzheimer’s [190] and chronic lung disease [191]. In view of this point, cromolyn may be useful against COVID-19; a clinical trial has been initiated to study the combination of this compound with standard treatment in patients with COVID-19 pneumonia (NCT05077917).

**Targeting the PD-1/PD-L1 axis**—The first clinical application of anti-PD-1 in cancer has represented a true breakthrough [192] and, since then, the PD-1/PD-L1 axis blockade has become an approved therapeutic strategy in several cancer types, representing an unprecedented advancement in cancer treatment, as single agents or in combination with other anticancer agents [193]. Moreover, in recent years, immune checkpoint blockade has progressively been moved from late disease stages to the first-line setting, acknowledging its dramatic impact on patients’ prognosis in various cancer types, among which non-small cell lung cancer [194], melanoma [195], urothelial carcinoma [196] represent pioneering settings. As above mentioned, the PD-1/PD-L1 axis is a major player in immune exhaustion in COVID-19 infection, and lymphocytic PD-1 expression levels correlate with disease severity in patients affected by SARS-CoV-2. This evidence, directly derived from early findings and studies on lymphopenic patients during the COVID-19 pandemic, has prompted a search for potential therapeutic benefits derived from PD-1/PD-L1 axis blockade. Indeed, three clinical trials have investigated the role of the anti PD-1 nivolumab in SARS-CoV-2 patients, but the results are not available yet (NCT04413838; NCT04343144; NCT04356508). Other immune checkpoints have been implicated in immune exhaustion in COVID-19, such as TIM-3 and LAG-3 [197], but no dedicated clinical trial has been developed so far.

**Targeting PARP enzymes**—While PARP inhibition is a therapeutic option in an increasing number of cancer histotypes [138], the “repurposing” of PARP inhibitors in the context of COVID-19 infection, with the aim of tapering overzealous immune responses and their potentially detrimental effects, has been hypothesized, but has not yet been tested in the clinic [198]. Moreover, given the mono-ADP-ribosyl hydrolase activity exerted by SARS-CoV-2, the utility of PARP inhibition (and its timing with respect to early vs. advanced infection) in the treatment of COVID-19 remains to be determined, given the delicate balance between efficacious vs. overzealous immune activation and the multifaceted role of PARP enzymes in this context. Indeed, a completely different therapeutic strategy linked to PARP signaling has also been proposed, suggesting that NAD-enhancing strategies could boost PARP antiviral activity, hindering SARS-CoV-2 infection progression [199]. Hence, the double-faced role of PARPs in COVID-19 suggests a cautious approach towards the potential repurposing of PARP inhibitors in COVID-19 treatment.

In Table 1, the most relevant ongoing observational and interventional trials, addressing the intersections of COVID-19 and cancer, are listed.

## 6. Conclusions

In this review, we have highlighted the immunobiological connections shared by SARS-CoV-2 infection and cancer. In particular, we have underlined the dynamic involvement of different cytokines and immune system pathways at different disease stages in both conditions, and pinpointed the most relevant therapeutic intersections. These interdisciplinary considerations warrant attention for the development of future research avenues, which could take into account the clinical challenges stemming from enduring/frustrated chronic immune responses in COVID-19 and cancer, to develop novel therapeutic strategies to tackle the chronicization of immune responses in both disease settings.

## Figures and Tables

**Table 1:** Selected ongoing studies on COVID-19 and cancer (interventional and observational). Source: clinicaltrials.gov. accessed on 2 October 2022.

Study Title	Study Type	Interventions	Phase	Clinical Trial Identifier
COVID-19 Prevention and Treatment in Cancer; a Sequential Multiple Assignment Randomized Trial; (C-SMART)	Interventional	Drug: interferon alfa Drug: selinexor Drug: lenzilumab	III	NCT04534725
Rintatolimod and IFN Alpha-2b for the Treatment of COVID-19 in Cancer Patients	Interventional	Other: best practice Biological: recombinant interferon alfa-2b Drug: rintatolimod	I///II	NCT04379518
Leflunomide for the Treatment of Severe COVID-19 in Patients With a Concurrent Malignancy	Interventional	Other: best practice Drug: leflunomide Drug: placebo administration	I///II	NCT04532372
Viral Specific T Cell Therapy for COVID-19 Related Pneumonia in Cancer Patients	Interventional	Biological: SARS-CoV-2 antigen-specific cytotoxic T-lymphocytes	I	NCT04742595
A Trial of the Safety and Immunogenicity of the COVID-19 Vaccine (mRNA-1273) in Participants With Hematologic Malignancies and Various Regimens of Immunosuppression, and in Participants With Solid Tumors on PD1/PDL1 Inhibitor Therapy, Including Booster Doses of Vaccine	Interventional	Biological: mRNA 1273 injection	II	NCT04847050
(COVID-19) Longitudinal Neutralizing Antibody Titers in Cancer Patients Receiving Different Anticancer Therapies	Observational	-	-	NCT05384509
Antibodies Production After COVID-19 Vaccination Among Patients with Medical History of Cancer and Anti-CD-20 Treatment	Observational	-	-	NCT04779996
Immunity Against Severe Acute Respiratory Syndrome Coronavirus 2 Disease (COVID-19] in the Oncology Outpatient Setting (COVIDOUT)	Observational	-	-	NCT04779346
A Study on the Immune Response to COVID-19 Vaccination in Cancer Patients—the IOSI-COVID-19-001 Study	Observational	-	-	NCT04800146
ASCO Survey on COVID-19 in Oncology (ASCO) Registry	Observational	-	-	NCT04659135
Investigation of the B- and T-cell Repertoire and Immune Response in Patients with Acute and Resolved COVID-19 Infection	Observational	-	-	NCT04362865

## Data Availability

Data sharing not applicable.

## References

[1] Scagliotti G., Novello S., Veltri A., Boccuzzi A., Perboni A., Terzolo M. (2020). Patients With Lung Cancer and Coronavirus Disease 2019 Epidemic: An Experience From an Italian University Hospital. JTO Clin. Res. Rep..

[2] Smeltzer M.P., Scagliotti G.V., Wakelee H.A., Mitsudomi T., Roy U.B., Clark R.C., Arndt R., Pruett C.D., Kelly K.L., Ujhazy P. (2022). International Association for the Study of Lung Cancer Study of the Impact of Coronavirus Disease 2019 on International Lung Cancer Clinical Trials. J. Thorac. Oncol..

[3] Richards M., Anderson M., Carter P., Ebert B.L., Mossialos E. (2020). The impact of the COVID-19 pandemic on cancer care. Nat. Cancer.

[4] Lee L.Y., Cazier J.B., Angelis V., Arnold R., Bisht V., Campton N.A., Chackathayil J., Cheng V.W., Curley H.M., Fittall M.W. (2020). COVID-19 mortality in patients with cancer on chemotherapy or other anticancer treatments: A prospective cohort study. Lancet.

[5] Kuderer N.M., Choueiri T.K., Shah D.P., Shyr Y., Rubinstein S.M., Rivera D.R., Shete S., Hsu C.Y., Desai A., de Lima Lopes G. (2020). Clinical impact of COVID-19 on patients with cancer (CCC19): A cohort study. Lancet.

[6] Yuan Y., Cao D., Zhang Y., Ma J., Qi J., Wang Q., Lu G., Wu Y., Yan J., Shi Y. (2017). Cryo-EM structures of MERS-CoV and SARS-CoV spike glycoproteins reveal the dynamic receptor binding domains. Nat. Commun..

[7] Walls A.C., Park Y.J., Tortorici M.A., Wall A., McGuire A.T., Veesler D. (2020). Structure, Function, and Antigenicity of the SARS-CoV-2 Spike Glycoprotein. Cell.

[8] Hui K.P.Y., Cheung M.C., Perera R.A.P.M., Ng K.C., Bui C.H.T., Ho J.C.W., Ng M.M.T., Kuok D.I.T., Shih K.C., Tsao S.W. (2020). Tropism, replication competence, and innate immune responses of the coronavirus SARS-CoV-2 in human respiratory tract and conjunctiva: An analysis in ex-vivo and in-vitro cultures. Lancet Respir. Med..

[9] Mathew D., Giles J.R., Baxter A.E., Oldridge D.A., Greenplate A.R., Wu J.E., Alanio C., Kuri-Cervantes L., Pampena M.B., D’Andrea K. (2020). Deep immune profiling of COVID-19 patients reveals distinct immunotypes with therapeutic implications. Science.

[10] Gold M.S., Sehayek D., Gabrielli S., Zhang X., McCusker C., Ben-Shoshan M. (2020). COVID-19 and comorbidities: A systematic review and meta-analysis. Postgrad. Med..

[11] Chavez-MacGregor M., Lei X., Zhao H., Scheet P., Giordano S.H. (2022). Evaluation of COVID-19 Mortality and Adverse Outcomes in US Patients With or Without Cancer. JAMA Oncol..

[12] Marfella R., Sardu C., D’Onofrio N., Prattichizzo F., Scisciola L., Messina V., La Grotta R., Balestrieri M.L., Maggi P., Napoli C. (2022). Glycaemic control is associated with SARS-CoV-2 breakthrough infections in vaccinated patients with type 2 diabetes. Nat. Commun..

[13] Marfella R., D’Onofrio N., Sardu C., Scisciola L., Maggi P., Coppola N., Romano C., Messina V., Turriziani F., Siniscalchi M. (2022). Does poor glycaemic control affect the immunogenicity of the COVID-19 vaccination in patients with type 2 diabetes: The CAVEAT study. Diabetes. Obes. Metab..

[14] Castelo-Branco L., Tsourti Z., Gennatas S., Rogado J., Sekacheva M., Viñal D., Lee R., Croitoru A., Vitorino M., Khallaf S. (2022). COVID-19 in patients with cancer: First report of the ESMO international, registry-based, cohort study (ESMO-CoCARE). ESMO Open.

[15] Lennon H., Sperrin M., Badrick E., Renehan A.G. (2016). The Obesity Paradox in Cancer: A Review. Curr. Oncol. Rep..

[16] Petrelli F., Cortellini A., Indini A., Tomasello G., Ghidini M., Nigro O., Salati M., Dottorini L., Iaculli A., Varricchio A. (2020). Obesity paradox in patients with cancer: A systematic review and meta-analysis of 6,320,365 patients. medRxiv.

[17] Romero Starke K., Reissig D., Petereit-Haack G., Schmauder S., Nienhaus A., Seidler A. (2021). The isolated effect of age on the risk of COVID-19 severe outcomes: A systematic review with meta-analysis. BMJ Glob. Health.

[18] Napoli C., Tritto I., Benincasa G., Mansueto G., Ambrosio G. (2020). Cardiovascular involvement during COVID-19 and clinical implications in elderly patients. A review. Ann. Med. Surg..

[19] Napoli C., Tritto I., Mansueto G., Coscioni E., Ambrosio G. (2020). Immunosenescence exacerbates the COVID-19. Arch Gerontol. Geriatr..

[20] Sciacchitano S., De Vitis C., D’Ascanio M., Giovagnoli S., De Dominicis C., Laghi A., Anibaldi P., Petrucca A., Salerno G., Santino I. (2021). Gene signature and immune cell profiling by high-dimensional, single-cell analysis in COVID-19 patients, presenting Low T3 syndrome and coexistent hematological malignancies. J. Transl. Med..

[21] Montisci A., Vietri M.T., Palmieri V., Sala S., Donatelli F., Napoli C. (2021). Cardiac Toxicity Associated with Cancer Immunotherapy and Biological Drugs. Cancers.

[22] Montisci A., Palmieri V., Liu J.E., Vietri M.T., Cirri S., Donatelli F., Napoli C. (2021). Severe Cardiac Toxicity Induced by Cancer Therapies Requiring Intensive Care Unit Admission. Front. Cardiovasc. Med..

[23] Bracci L., Schiavoni G., Sistigu A., Belardelli F. (2014). Immune-based mechanisms of cytotoxic chemotherapy: Implications for the design of novel and rationale-based combined treatments against cancer. Cell Death Differ..

[24] Zitvogel L., Apetoh L., Ghiringhelli F., Kroemer G. (2008). Immunological aspects of cancer chemotherapy. Nat. Rev. Immunol..

[25] Galluzzi L., Zitvogel L., Kroemer G. (2016). Immunological Mechanisms Underneath the Efficacy of Cancer Therapy. Cancer Immunol. Res..

[26] Galluzzi L., Buqué A., Kepp O., Zitvogel L., Kroemer G. (2015). Immunological Effects of Conventional Chemotherapy and Targeted Anticancer Agents. Cancer Cell.

[27] Rasmussen L., Arvin A. (1982). Chemotherapy-induced immunosuppression. Environ. Health Perspect..

[28] Mansueto G., Niola M., Napoli C. (2020). Can COVID 2019 induce a specific cardiovascular damage or it exacerbates pre-existing cardiovascular diseases?. Pathol. Res. Pract..

[29] Xie Y., Xu E., Bowe B., Al-Aly Z. (2022). Long-term cardiovascular outcomes of COVID-19. Nat. Med..

[30] Zheng Y.Y., Ma Y.T., Zhang J.Y., Xie X. (2020). COVID-19 and the cardiovascular system. Nat. Rev. Cardiol..

[31] Menna P., Paz O.G., Chello M., Covino E., Salvatorelli E., Minotti G. (2012). Anthracycline cardiotoxicity. Expert. Opin. Drug. Saf..

[32] Pondé N.F., Lambertini M., de Azambuja E. (2016). Twenty years of anti-HER2 therapy-associated cardiotoxicity. ESMO Open.

[33] Furlan A., Forner G., Cipriani L., Vian E., Rigoli R., Gherlinzoni F., Scotton P. (2021). COVID-19 in B Cell-Depleted Patients After Rituximab: A Diagnostic and Therapeutic Challenge. Front. Immunol..

[34] Boekel L., Wolbink G.J. (2022). Rituximab during the COVID-19 pandemic: Time to discuss treatment options with patients. Lancet Rheumatol..

[35] Yu M., Cheng Y., Zhang R., Wen T., Huai S., Wei X., Zhang L. (2022). Evaluating treatment strategies for non-small cell lung cancer during COVID-19: A propensity score matching analysis. Medicine.

[36] Turnquist C., Ryan B.M., Horikawa I., Harris B.T., Harris C.C. (2020). Cytokine Storms in Cancer and COVID-19. Cancer Cell.

[37] Shalapour S., Karin M. (2015). Immunity, inflammation, and cancer: An eternal fight between good and evil. J. Clin. Investig..

[38] Germolec D.R., Shipkowski K.A., Frawley R.P., Evans E. (2018). Markers of Inflammation. Methods Mol. Biol..

[39] Chen L., Deng H., Cui H., Fang J., Zuo Z., Deng J., Li Y., Wang X., Zhao L. (2018). Inflammatory responses and inflammation-associated diseases in organs. Oncotarget.

[40] Altmann D.M., Boyton R.J. (2020). SARS-CoV-2 T cell immunity: Specificity, function, durability, and role in protection. Sci. Immunol..

[41] Napoli C., Benincasa G., Criscuolo C., Faenza M., Liberato C., Rusciano M. (2021). Immune reactivity during COVID-19: Implications for treatment. Immunol. Lett..

[42] Blanco-Melo D., Nilsson-Payant B.E., Liu W.C., Uhl S., Hoagland D., Møller R., Jordan T.X., Oishi K., Panis M., Sachs D. (2020). Imbalanced Host Response to SARS-CoV-2 Drives Development of COVID-19. Cell.

[43] Hadjadj J., Yatim N., Barnabei L., Corneau A., Boussier J., Smith N., Péré H., Charbit B., Bondet V., Chenevier-Gobeaux C. (2020). Impaired type I interferon activity and inflammatory responses in severe COVID-19 patients. Science.

[44] Rydyznski Moderbacher C., Ramirez S.I., Dan J.M., Grifoni A., Hastie K.M., Weiskopf D., Belanger S., Abbott R.K., Kim C., Choi J. (2020). Antigen-Specific Adaptive Immunity to SARS-CoV-2 in Acute COVID-19 and Associations with Age and Disease Severity. Cell.

[45] Sette A., Crotty S. (2021). Adaptive immunity to SARS-CoV-2 and COVID-19. Cell.

[46] Prager I., Watzl C. (2019). Mechanisms of natural killer cell-mediated cellular cytotoxicity. J. Leukoc. Biol..

[47] Janeway C.A. (2001). How the immune system protects the host from infection. Microbes. Infect..

[48] Kim K.D., Zhao J., Auh S., Yang X., Du P., Tang H., Fu Y.X. (2007). Adaptive immune cells temper initial innate responses. Nat. Med..

[49] Jordan S.C. (2021). Innate and adaptive immune responses to SARS-CoV-2 in humans: Relevance to acquired immunity and vaccine responses. Clin. Exp. Immunol..

[50] Grifoni A., Weiskopf D., Ramirez S.I., Mateus J., Dan J.M., Moderbacher C.R., Rawlings S.A., Sutherland A., Premkumar L., Jadi R.S. (2020). Targets of T Cell Responses to SARS-CoV-2 Coronavirus in Humans with COVID-19 Disease and Unexposed Individuals. Cell.

[51] Sekine T., Perez-Potti A., Rivera-Ballesteros O., Strålin K., Gorin J.B., Olsson A., Llewellyn-Lacey S., Kamal H., Bogdanovic G., Muschiol S. (2020). Robust T Cell Immunity in Convalescent Individuals with Asymptomatic or Mild COVID-19. Cell.

[52] Liao M., Liu Y., Yuan J., Wen Y., Xu G., Zhao J., Cheng L., Li J., Wang X., Wang F. (2020). Single-cell landscape of bronchoalveolar immune cells in patients with COVID-19. Nat. Med..

[53] Zhou R., To K.K., Wong Y.C., Liu L., Zhou B., Li X., Huang H., Mo Y., Luk T.Y., Lau T.T. (2020). Acute SARS-CoV-2 Infection Impairs Dendritic Cell and T Cell Responses. Immunity.

[54] Bergamaschi L., Mescia F., Turner L., Hanson A.L., Kotagiri P., Dunmore B.J., Ruffieux H., De Sa A., Huhn O., Morgan M.D. (2021). Longitudinal analysis reveals that delayed bystander CD8+ T cell activation and early immune pathology distinguish severe COVID-19 from mild disease. Immunity.

[55] Notarbartolo S., Ranzani V., Bandera A., Gruarin P., Bevilacqua V., Putignano A.R., Gobbini A., Galeota E., Manara C., Bombaci M. (2021). Integrated longitudinal immunophenotypic, transcriptional and repertoire analyses delineate immune responses in COVID-19 patients. Sci. Immunol..

[56] Braun J., Loyal L., Frentsch M., Wendisch D., Georg P., Kurth F., Hippenstiel S., Dingeldey M., Kruse B., Fauchere F. (2020). SARS-CoV-2-reactive T cells in healthy donors and patients with COVID-19. Nature.

[57] Tan A.T., Linster M., Tan C.W., Le Bert N., Chia W.N., Kunasegaran K., Zhuang Y., Tham C.Y.L., Chia A., Smith G.J.D. (2021). Early induction of functional SARS-CoV-2-specific T cells associates with rapid viral clearance and mild disease in COVID-19 patients. Cell Rep..

[58] Schulien I., Kemming J., Oberhardt V., Wild K., Seidel L.M., Killmer S., Sagar, Daul F., Salvat Lago M., Decker A. (2021). Characterization of pre-existing and induced SARS-CoV-2-specific CD8. Nat. Med..

[59] Grimaldi V., Benincasa G., Moccia G., Sansone A., Signoriello G., Napoli C. (2022). Evaluation of circulating leucocyte populations both in subjects with previous SARS-CoV-2 infection and in healthy subjects after vaccination. J. Immunol. Methods.

[60] Qin C., Zhou L., Hu Z., Zhang S., Yang S., Tao Y., Xie C., Ma K., Shang K., Wang W. (2020). Dysregulation of Immune Response in Patients With Coronavirus 2019 (COVID-19) in Wuhan, China. Clin Infect. Dis..

[61] Stephen-Victor E., Das M., Karnam A., Pitard B., Gautier J.F., Bayry J. (2020). Potential of regulatory T-cell-based therapies in the management of severe COVID-19. Eur. Respir. J..

[62] Neumann J., Prezzemolo T., Vanderbeke L., Roca C.P., Gerbaux M., Janssens S., Willemsen M., Burton O., Van Mol P., Van Herck Y. (2020). Increased IL-10-producing regulatory T cells are characteristic of severe cases of COVID-19. Clin. Transl. Immunol..

[63] Tan M., Liu Y., Zhou R., Deng X., Li F., Liang K., Shi Y. (2020). Immunopathological characteristics of coronavirus disease 2019 cases in Guangzhou, China. Immunology.

[64] Virgin H.W., Wherry E.J., Ahmed R. (2009). Redefining chronic viral infection. Cell.

[65] Huang C., Wang Y., Li X., Ren L., Zhao J., Hu Y., Zhang L., Fan G., Xu J., Gu X. (2020). Clinical features of patients infected with 2019 novel coronavirus in Wuhan, China. Lancet.

[66] McGonagle D., Sharif K., O’Regan A., Bridgewood C. (2020). The Role of Cytokines including Interleukin-6 in COVID-19 induced Pneumonia and Macrophage Activation Syndrome-Like Disease. Autoimmun. Rev..

[67] Murthy H., Iqbal M., Chavez J.C., Kharfan-Dabaja M.A. (2019). Cytokine Release Syndrome: Current Perspectives. Immunotargets Ther..

[68] Behrens E.M., Koretzky G.A. (2017). Review: Cytokine Storm Syndrome: Looking Toward the Precision Medicine Era. Arthritis Rheumatol..

[69] Del Valle D.M., Kim-Schulze S., Huang H.H., Beckmann N.D., Nirenberg S., Wang B., Lavin Y., Swartz T.H., Madduri D., Stock A. (2020). An inflammatory cytokine signature predicts COVID-19 severity and survival. Nat. Med..

[70] Chen G., Wu D., Guo W., Cao Y., Huang D., Wang H., Wang T., Zhang X., Chen H., Yu H. (2020). Clinical and immunological features of severe and moderate coronavirus disease 2019. J Clin Invest.

[71] Liu Y., Zhang C., Huang F., Yang Y., Wang F., Yuan J., Zhang Z., Qin Y., Li X., Zhao D. (2020). Elevated plasma levels of selective cytokines in COVID-19 patients reflect viral load and lung injury. Natl. Sci. Rev..

[72] Narazaki M., Kishimoto T. (2018). The Two-Faced Cytokine IL-6 in Host Defense and Diseases. Int. J. Mol. Sci..

[73] Arnaldez F.I., O’Day S.J., Drake C.G., Fox B.A., Fu B., Urba W.J., Montesarchio V., Weber J.S., Wei H., Wigginton J.M. (2020). The Society for Immunotherapy of Cancer perspective on regulation of interleukin-6 signaling in COVID-19-related systemic inflammatory response. J. Immunother. Cancer.

[74] Kumari N., Dwarakanath B.S., Das A., Bhatt A.N. (2016). Role of interleukin-6 in cancer progression and therapeutic resistance. Tumour. Biol..

[75] Johnson D.E., O’Keefe R.A., Grandis J.R. (2018). Targeting the IL-6/JAK/STAT3 signalling axis in cancer. Nat. Rev. Clin. Oncol..

[76] Liu Z., Li J., Chen D., Gao R., Zeng W., Chen S., Huang Y., Huang J., Long W., Li M. (2020). Dynamic Interleukin-6 Level Changes as a Prognostic Indicator in Patients With COVID-19. Front. Pharmacol..

[77] Mehta A.K., Gracias D.T., Croft M. (2018). TNF activity and T cells. Cytokine.

[78] Pasquereau S., Kumar A., Herbein G. (2017). Targeting TNF and TNF Receptor Pathway in HIV-1 Infection: From Immune Activation to Viral Reservoirs. Viruses.

[79] Popivanova B.K., Kitamura K., Wu Y., Kondo T., Kagaya T., Kaneko S., Oshima M., Fujii C., Mukaida N. (2008). Blocking TNF-alpha in mice reduces colorectal carcinogenesis associated with chronic colitis. J. Clin. Investig..

[80] Zhaorigetu S., Yanaka N., Sasaki M., Watanabe H., Kato N. (2003). Silk protein, sericin, suppresses DMBA-TPA-induced mouse skin tumorigenesis by reducing oxidative stress, inflammatory responses and endogenous tumor promoter TNF-alpha. Oncol. Rep..

[81] Berberoglu U., Yildirim E., Celen O. (2004). Serum levels of tumor necrosis factor alpha correlate with response to neoadjuvant chemotherapy in locally advanced breast cancer. Int. J. Biol. Markers.

[82] Michalaki V., Syrigos K., Charles P., Waxman J. (2004). Serum levels of IL-6 and TNF-alpha correlate with clinicopathological features and patient survival in patients with prostate cancer. Br. J. Cancer.

[83] Ferrajoli A., Keating M.J., Manshouri T., Giles F.J., Dey A., Estrov Z., Koller C.A., Kurzrock R., Thomas D.A., Faderl S. (2002). The clinical significance of tumor necrosis factor-alpha plasma level in patients having chronic lymphocytic leukemia. Blood.

[84] Szlosarek P.W., Grimshaw M.J., Kulbe H., Wilson J.L., Wilbanks G.D., Burke F., Balkwill F.R. (2006). Expression and regulation of tumor necrosis factor alpha in normal and malignant ovarian epithelium. Mol. Cancer Ther..

[85] Mulchandani R., Lyngdoh T., Kakkar A.K. (2021). Deciphering the COVID-19 cytokine storm: Systematic review and meta-analysis. Eur. J. Clin. Investig..

[86] Pestka S., Krause C.D., Walter M.R. (2004). Interferons, interferon-like cytokines, and their receptors. Immunol. Rev..

[87] Lee A.J., Ashkar A.A. (2018). The Dual Nature of Type I and Type II Interferons. Front. Immunol..

[88] Witte K., Witte E., Sabat R., Wolk K. (2010). IL-28A, IL-28B, and IL-29: Promising cytokines with type I interferon-like properties. Cytokine Growth Factor Rev..

[89] Snell L.M., McGaha T.L., Brooks D.G. (2017). Type I Interferon in Chronic Virus Infection and Cancer. Trends Immunol..

[90] McNab F., Mayer-Barber K., Sher A., Wack A., O’Garra A. (2015). Type I interferons in infectious disease. Nat. Rev. Immunol..

[91] Musella M., Manic G., De Maria R., Vitale I., Sistigu A. (2017). Type-I-interferons in infection and cancer: Unanticipated dynamics with therapeutic implications. Oncoimmunology.

[92] Aricò E., Castiello L., Capone I., Gabriele L., Belardelli F. (2019). Type I Interferons and Cancer: An Evolving Story Demanding Novel Clinical Applications. Cancers.

[93] Schreiber R.D., Old L.J., Smyth M.J. (2011). Cancer immunoediting: Integrating immunity’s roles in cancer suppression and promotion. Science.

[94] Zitvogel L., Galluzzi L., Kepp O., Smyth M.J., Kroemer G. (2015). Type I interferons in anticancer immunity. Nat. Rev. Immunol..

[95] Gupta R. (2020). The double edged interferon riddle in COVID-19 pathogenesis. Crit. Care.

[96] Lopez L., Sang P.C., Tian Y., Sang Y. (2020). Dysregulated Interferon Response Underlying Severe COVID-19. Viruses.

[97] Israelow B., Song E., Mao T., Lu P., Meir A., Liu F., Alfajaro M.M., Wei J., Dong H., Homer R.J. (2020). Mouse model of SARS-CoV-2 reveals inflammatory role of type I interferon signaling. J. Exp. Med..

[98] Liu T., Zhang L., Joo D., Sun S.C. (2017). NF-κB signaling in inflammation. Signal Transduct. Target. Ther..

[99] Oeckinghaus A., Hayden M.S., Ghosh S. (2011). Crosstalk in NF-κB signaling pathways. Nat. Immunol..

[100] Yu H., Lin L., Zhang Z., Zhang H., Hu H. (2020). Targeting NF-κB pathway for the therapy of diseases: Mechanism and clinical study. Signal Transduct. Target. Ther..

[101] Moynagh P.N. (2005). The NF-kappaB pathway. J Cell Sci.

[102] Neufeldt C.J., Cerikan B., Cortese M., Frankish J., Lee J.Y., Plociennikowska A., Heigwer F., Prasad V., Joecks S., Burkart S.S. (2022). SARS-CoV-2 infection induces a pro-inflammatory cytokine response through cGAS-STING and NF-κB. Commun. Biol..

[103] Su C.M., Wang L., Yoo D. (2021). Activation of NF-κB and induction of proinflammatory cytokine expressions mediated by ORF7a protein of SARS-CoV-2. Sci. Rep..

[104] Catrysse L., van Loo G. (2017). Inflammation and the Metabolic Syndrome: The Tissue-Specific Functions of NF-κB. Trends Cell Biol..

[105] Apicella M., Campopiano M.C., Mantuano M., Mazoni L., Coppelli A., Del Prato S. (2020). COVID-19 in people with diabetes: Understanding the reasons for worse outcomes. Lancet Diabetes Endocrinol..

[106] Popkin B.M., Du S., Green W.D., Beck M.A., Algaith T., Herbst C.H., Alsukait R.F., Alluhidan M., Alazemi N., Shekar M. (2020). Individuals with obesity and COVID-19: A global perspective on the epidemiology and biological relationships. Obes. Rev..

[107] Gilmore T.D. (1999). Multiple mutations contribute to the oncogenicity of the retroviral oncoprotein v-Rel. Oncogene.

[108] Gilmore T.D., Kalaitzidis D., Liang M.C., Starczynowski D.T. (2004). The c-Rel transcription factor and B-cell proliferation: A deal with the devil. Oncogene.

[109] Neri A., Chang C.C., Lombardi L., Salina M., Corradini P., Maiolo A.T., Chaganti R.S., Dalla-Favera R. (1991). B cell lymphoma-associated chromosomal translocation involves candidate oncogene lyt-10, homologous to NF-kappa B p50. Cell.

[110] Bredel M., Scholtens D.M., Yadav A.K., Alvarez A.A., Renfrow J.J., Chandler J.P., Yu I.L., Carro M.S., Dai F., Tagge M.J. (2011). NFKBIA deletion in glioblastomas. N. Engl. J. Med..

[111] Greenman C., Stephens P., Smith R., Dalgliesh G.L., Hunter C., Bignell G., Davies H., Teague J., Butler A., Stevens C. (2007). Patterns of somatic mutation in human cancer genomes. Nature.

[112] Pflueger D., Terry S., Sboner A., Habegger L., Esgueva R., Lin P.C., Svensson M.A., Kitabayashi N., Moss B.J., MacDonald T.Y. (2011). Discovery of non-ETS gene fusions in human prostate cancer using next-generation RNA sequencing. Genome Res..

[113] Boehm J.S., Zhao J.J., Yao J., Kim S.Y., Firestein R., Dunn I.F., Sjostrom S.K., Garraway L.A., Weremowicz S., Richardson A.L. (2007). Integrative genomic approaches identify IKBKE as a breast cancer oncogene. Cell.

[114] Terzić J., Grivennikov S., Karin E., Karin M. (2010). Inflammation and colon cancer. Gastroenterology.

[115] Pikarsky E., Porat R.M., Stein I., Abramovitch R., Amit S., Kasem S., Gutkovich-Pyest E., Urieli-Shoval S., Galun E., Ben-Neriah Y. (2004). NF-kappaB functions as a tumour promoter in inflammation-associated cancer. Nature.

[116] Hagemann T., Lawrence T., McNeish I., Charles K.A., Kulbe H., Thompson R.G., Robinson S.C., Balkwill F.R. (2008). “Re-educating” tumor-associated macrophages by targeting NF-kappaB. J. Exp. Med..

[117] Darnell J.E. (1997). STATs and gene regulation. Science.

[118] O’Shea J.J., Schwartz D.M., Villarino A.V., Gadina M., McInnes I.B., Laurence A. (2015). The JAK-STAT pathway: Impact on human disease and therapeutic intervention. Annu. Rev. Med..

[119] Owen K.L., Brockwell N.K., Parker B.S. (2019). JAK-STAT Signaling: A Double-Edged Sword of Immune Regulation and Cancer Progression. Cancers.

[120] Aittomäki S., Pesu M. (2014). Therapeutic targeting of the Jak/STAT pathway. Basic Clin. Pharmacol. Toxicol..

[121] Vainchenker W., Constantinescu S.N. (2013). JAK/STAT signaling in hematological malignancies. Oncogene.

[122] Vainchenker W., Delhommeau F., Constantinescu S.N., Bernard O.A. (2011). New mutations and pathogenesis of myeloproliferative neoplasms. Blood.

[123] Pardanani A., Lasho T.L., Finke C., Hanson C.A., Tefferi A. (2007). Prevalence and clinicopathologic correlates of JAK2 exon 12 mutations in JAK2V617F-negative polycythemia vera. Leukemia.

[124] Koskela H.L., Eldfors S., Ellonen P., van Adrichem A.J., Kuusanmäki H., Andersson E.I., Lagström S., Clemente M.J., Olson T., Jalkanen S.E. (2012). Somatic STAT3 mutations in large granular lymphocytic leukemia. N. Engl. J. Med..

[125] Xu Y.H., Lu S. (2014). A meta-analysis of STAT3 and phospho-STAT3 expression and survival of patients with non-small-cell lung cancer. Eur. J. Surg. Oncol..

[126] Mirtti T., Leiby B.E., Abdulghani J., Aaltonen E., Pavela M., Mamtani A., Alanen K., Egevad L., Granfors T., Josefsson A. (2013). Nuclear Stat5a/b predicts early recurrence and prostate cancer-specific death in patients treated by radical prostatectomy. Hum. Pathol..

[127] Macha M.A., Matta A., Kaur J., Chauhan S.S., Thakar A., Shukla N.K., Gupta S.D., Ralhan R. (2011). Prognostic significance of nuclear pSTAT3 in oral cancer. Head Neck..

[128] Messina J.L., Yu H., Riker A.I., Munster P.N., Jove R.L., Daud A.I. (2008). Activated stat-3 in melanoma. Cancer Control.

[129] Sonnenblick A., Uziely B., Nechushtan H., Kadouri L., Galun E., Axelrod J.H., Katz D., Daum H., Hamburger T., Maly B. (2013). Tumor STAT3 tyrosine phosphorylation status, as a predictor of benefit from adjuvant chemotherapy for breast cancer. Breast Cancer Res. Treat..

[130] Kusaba T., Nakayama T., Yamazumi K., Yakata Y., Yoshizaki A., Inoue K., Nagayasu T., Sekine I. (2006). Activation of STAT3 is a marker of poor prognosis in human colorectal cancer. Oncol. Rep..

[131] Chen D.Y., Khan N., Close B.J., Goel R.K., Blum B., Tavares A.H., Kenney D., Conway H.L., Ewoldt J.K., Chitalia V.C. (2021). SARS-CoV-2 Disrupts Proximal Elements in the JAK-STAT Pathway. J. Virol..

[132] Huang P.W., Chang J.W. (2019). Immune checkpoint inhibitors win the 2018 Nobel Prize. Biomed. J..

[133] Gajewski T.F., Schreiber H., Fu Y.X. (2013). Innate and adaptive immune cells in the tumor microenvironment. Nat. Immunol..

[134] Zhao Q., Meng M., Kumar R., Wu Y., Huang J., Deng Y., Weng Z., Yang L. (2020). Lymphopenia is associated with severe coronavirus disease 2019 (COVID-19) infections: A systemic review and meta-analysis. Int. J. Infect. Dis..

[135] Diao B., Wang C., Tan Y., Chen X., Liu Y., Ning L., Chen L., Li M., Wang G., Yuan Z. (2020). Reduction and Functional Exhaustion of T Cells in Patients With Coronavirus Disease 2019 (COVID-19). Front. Immunol..

[136] Sabbatino F., Conti V., Franci G., Sellitto C., Manzo V., Pagliano P., De Bellis E., Masullo A., Salzano F.A., Caputo A. (2021). PD-L1 Dysregulation in COVID-19 Patients. Front. Immunol..

[137] Kraus W.L. (2015). PARPs and ADP-Ribosylation: 50 Years… and Counting. Mol. Cell.

[138] Grignani G., Merlini A., Sangiolo D., D’Ambrosio L., Pignochino Y. (2020). Delving into PARP inhibition from bench to bedside and back. Pharmacol. Ther..

[139] Alhammad Y.M.O., Kashipathy M.M., Roy A., Gagné J.-P., McDonald P., Gao P., Nonfoux L., Battaile K.P., Johnson D.K., Holmstrom E.D. (2021). The SARS-CoV-2 Conserved Macrodomain Is a Mono-ADP-Ribosylhydrolase. J. Virol..

[140] Fehr A.R., Singh S.A., Kerr C.M., Mukai S., Higashi H., Aikawa M. (2020). The impact of PARPs and ADP-ribosylation on inflammation and host-pathogen interactions. Genes Dev..

[141] Cohen M.S. (2020). Interplay between compartmentalized NAD. Genes Dev..

[142] Hottiger M.O. (2015). Nuclear ADP-Ribosylation and Its Role in Chromatin Plasticity, Cell Differentiation, and Epigenetics. Annu. Rev. Biochem..

[143] Ryu K.W., Kim D.S., Kraus W.L. (2015). New facets in the regulation of gene expression by ADP-ribosylation and poly(ADP-ribose) polymerases. Chem. Rev..

[144] Gupte R., Liu Z., Kraus W.L. (2017). PARPs and ADP-ribosylation: Recent advances linking molecular functions to biological outcomes. Genes Dev..

[145] Lord C.J., Ashworth A. (2017). PARP inhibitors: Synthetic lethality in the clinic. Science.

[146] Lippitz B.E. (2013). Cytokine patterns in patients with cancer: A systematic review. Lancet Oncol..

[147] Salesi M., Shojaie B., Farajzadegan Z., Salesi N., Mohammadi E. (2021). TNF-α Blockers Showed Prophylactic Effects in Preventing COVID-19 in Patients with Rheumatoid Arthritis and Seronegative Spondyloarthropathies: A Case-Control Study. Rheumatol. Ther..

[148] Wise J. (2021). COVID-19: Arthritis drugs improve survival in intensive care patients, shows study. BMJ.

[149] Quesada J.R., Reuben J., Manning J.T., Hersh E.M., Gutterman J.U. (1984). Alpha interferon for induction of remission in hairy-cell leukemia. N. Engl. J. Med..

[150] Foon K.A., Sherwin S.A., Abrams P.G., Longo D.L., Fer M.F., Stevenson H.C., Ochs J.J., Bottino G.C., Schoenberger C.S., Zeffren J. (1984). Treatment of advanced non-Hodgkin’s lymphoma with recombinant leukocyte A interferon. N. Engl. J. Med..

[151] Eggermont A.M., Suciu S., Santinami M., Testori A., Kruit W.H., Marsden J., Punt C.J., Salès F., Gore M., MacKie R. (2008). Adjuvant therapy with pegylated interferon alfa-2b versus observation alone in resected stage III melanoma: Final results of EORTC 18991, a randomised phase III trial. Lancet.

[152] Smits E.L., Anguille S., Berneman Z.N. (2013). Interferon α may be back on track to treat acute myeloid leukemia. Oncoimmunology.

[153] Inoue M., Hisasue S., Nagae M., China T., Saito K., Isotani S., Yamaguchi R., Ide H., Muto S., Horie S. (2013). Interferon-α Treatment for Growing Teratoma Syndrome of the Testis. Case Rep. Nephrol. Urol..

[154] Radesi-Sarghi S., Arbion F., Dartigeas C., Delain M., Benboubker L., Hérault O., Colombat P., Gyan E. (2014). Interferon alpha with or without rituximab achieves a high response rate and durable responses in relapsed FL: 17 years’ experience in a single centre. Ann. Hematol..

[155] Monk P.D., Marsden R.J., Tear V.J., Brookes J., Batten T.N., Mankowski M., Gabbay F.J., Davies D.E., Holgate S.T., Ho L.P. (2021). Safety and efficacy of inhaled nebulised interferon beta-1a (SNG001) for treatment of SARS-CoV-2 infection: A randomised, double-blind, placebo-controlled, phase 2 trial. Lancet Respir. Med..

[156] Peiffer-Smadja N., Yazdanpanah Y. (2021). Nebulised interferon beta-1a for patients with COVID-19. Lancet Respir. Med..

[157] Nakhlband A., Fakhari A., Azizi H. (2021). Interferon-beta offers promising avenues to COVID-19 treatment: A systematic review and meta-analysis of clinical trial studies. Naunyn Schmiedebergs Arch Pharmacol..

[158] Wong C.K.H., Wan E.Y.F., Luo S., Ding Y., Lau E.H.Y., Ling P., Hu X., Lau E.C.H., Wong J., Zheng X. (2021). Clinical outcomes of different therapeutic options for COVID-19 in two Chinese case cohorts: A propensity-score analysis. eClinicalMedicine.

[159] Wang N., Zhan Y., Zhu L., Hou Z., Liu F., Song P., Qiu F., Wang X., Zou X., Wan D. (2020). Retrospective Multicenter Cohort Study Shows Early Interferon Therapy Is Associated with Favorable Clinical Responses in COVID-19 Patients. Cell Host Microbe.

[160] AIFA Medicine Usable for Treatment of COVID-19 Disease. https://www.aifa.gov.it/en/aggiornamento-sui-farmaci-utilizzabili-per-il-trattamento-della-malattia-covid19.

[161] Yanaihara N., Hirata Y., Yamaguchi N., Noguchi Y., Saito M., Nagata C., Takakura S., Yamada K., Okamoto A. (2016). Antitumor effects of interleukin-6 (IL-6)/interleukin-6 receptor (IL-6R) signaling pathway inhibition in clear cell carcinoma of the ovary. Mol. Carcinog..

[162] Goumas F.A., Holmer R., Egberts J.H., Gontarewicz A., Heneweer C., Geisen U., Hauser C., Mende M.M., Legler K., Röcken C. (2015). Inhibition of IL-6 signaling significantly reduces primary tumor growth and recurrencies in orthotopic xenograft models of pancreatic cancer. Int. J. Cancer.

[163] Grivennikov S., Karin E., Terzic J., Mucida D., Yu G.Y., Vallabhapurapu S., Scheller J., Rose-John S., Cheroutre H., Eckmann L. (2009). IL-6 and Stat3 are required for survival of intestinal epithelial cells and development of colitis-associated cancer. Cancer Cell.

[164] Sarfraz A., Sarfraz Z., Sarfraz M., Aftab H., Pervaiz Z. (2021). Tocilizumab and COVID-19: A meta-analysis of 2120 patients with severe disease and implications for clinical trial methodologies. Turk. J. Med. Sci..

[165] Durán-Méndez A., Aguilar-Arroyo A.D., Vivanco-Gómez E., Nieto-Ortega E., Pérez-Ortega D., Jiménez-Pérez C., Hernández-Skewes K.Y., Montiel-Bravo G., Roque-Reyes O.J., Romero-Lechuga F. (2021). Tocilizumab reduces COVID-19 mortality and pathology in a dose and timing-dependent fashion: A multi-centric study. Sci. Rep..

[166] Bose P., Verstovsek S. (2017). JAK2 inhibitors for myeloproliferative neoplasms: What is next?. Blood.

[167] Becker H., Engelhardt M., von Bubnoff N., Wäsch R. (2014). Ruxolitinib. Recent Results Cancer Res..

[168] Dougados M., van der Heijde D., Chen Y.C., Greenwald M., Drescher E., Liu J., Beattie S., Witt S., de la Torre I., Gaich C. (2017). Baricitinib in patients with inadequate response or intolerance to conventional synthetic DMARDs: Results from the RA-BUILD study. Ann. Rheum. Dis..

[169] Schwartz D.M., Bonelli M., Gadina M., O’Shea J.J. (2016). Type I/II cytokines, JAKs, and new strategies for treating autoimmune diseases. Nat. Rev. Rheumatol..

[170] WHO WHO Recommends Two New Drugs to Treat COVID-19. https://www.who.int/news/item/14-01-2022-who-recommends-two-new-drugs-to-treat-covid-19.

[171] Iastrebner M., Castro J., García Espina E., Lettieri C., Payaslian S., Cuesta M.C., Gutiérrez Fernández P., Mandrile A., Contreras A.P., Gervasoni S. (2021). Ruxolitinib in severe COVID-19: Results of a multicenter, prospective, single arm, open-label clinical study to investigate the efficacy and safety of ruxolitinib in patients with COVID-19 and severe acute respiratory syndrome. Rev. Fac. Cien. Med. Univ. Nac. Cordoba.

[172] Han M.K., Antila M., Ficker J.H., Gordeev I., Guerreros A., Bernus A.L., Roquilly A., Sifuentes-Osornio J., Tabak F., Teijeiro R. (2022). Ruxolitinib in addition to standard of care for the treatment of patients admitted to hospital with COVID-19 (RUXCOVID): A randomised, double-blind, placebo-controlled, phase 3 trial. Lancet Rheumatol..

[173] Marconi V.C., Ramanan A.V., de Bono S., Kartman C.E., Krishnan V., Liao R., Piruzeli M.L.B., Goldman J.D., Alatorre-Alexander J., de Cassia Pellegrini R. (2021). Efficacy and safety of baricitinib for the treatment of hospitalised adults with COVID-19 (COV-BARRIER): A randomised, double-blind, parallel-group, placebo-controlled phase 3 trial. Lancet Respir. Med..

[174] Lin Z., Niu J., Xu Y., Qin L., Ding J., Zhou L. (2022). Clinical efficacy and adverse events of baricitinib treatment for coronavirus disease-2019 (COVID-19): A systematic review and meta-analysis. J. Med. Virol..

[175] Iglesias Gómez R., Méndez R., Palanques-Pastor T., Ballesta-López O., Borrás Almenar C., Megías Vericat J.E., López-Briz E., Font-Noguera I., Menéndez Villanueva R., Román Iborra J.A. (2022). Baricitinib against severe COVID-19: Effectiveness and safety in hospitalised pretreated patients. Eur. J. Hosp. Pharm..

[176] Zidi I., Mestiri S., Bartegi A., Amor N.B. (2010). TNF-alpha and its inhibitors in cancer. Med. Oncol..

[177] Keystone E.C. (2003). Advances in targeted therapy: Safety of biological agents. Ann. Rheum. Dis..

[178] Askling J., Fored C.M., Baecklund E., Brandt L., Backlin C., Ekbom A., Sundström C., Bertilsson L., Cöster L., Geborek P. (2005). Haematopoietic malignancies in rheumatoid arthritis: Lymphoma risk and characteristics after exposure to tumour necrosis factor antagonists. Ann. Rheum. Dis..

[179] Haynes K., Beukelman T., Curtis J.R., Newcomb C., Herrinton L.J., Graham D.J., Solomon D.H., Griffin M.R., Chen L., Liu L. (2013). Tumor necrosis factor α inhibitor therapy and cancer risk in chronic immune-mediated diseases. Arthritis Rheum..

[180] Kokkotis G., Kitsou K., Xynogalas I., Spoulou V., Magiorkinis G., Trontzas I., Trontzas P., Poulakou G., Syrigos K., Bamias G. (2022). Systematic review with meta-analysis: COVID-19 outcomes in patients receiving anti-TNF treatments. Aliment. Pharmacol. Ther..

[181] Hachem H., Godara A., Schroeder C., Fein D., Mann H., Lawlor C., Marshall J., Klein A., Poutsiaka D., Breeze J.L. (2021). Rapid and sustained decline in CXCL-10 (IP-10) annotates clinical outcomes following TNF-α antagonist therapy in hospitalized patients with severe and critical COVID-19 respiratory failure. medRxiv.

[182] Horby P., Lim W.S., Emberson J.R., Mafham M., Bell J.L., Linsell L., Staplin N., Brightling C., Ustianowski A., Elmahi E. (2021). Dexamethasone in Hospitalized Patients with COVID-19. N. Engl. J. Med..

[183] Conforti F., Wang Y., Rodriguez J.A., Alberobello A.T., Zhang Y.W., Giaccone G. (2015). Molecular Pathways: Anticancer Activity by Inhibition of Nucleocytoplasmic Shuttling. Clin. Cancer Res..

[184] Chari A., Vogl D.T., Gavriatopoulou M., Nooka A.K., Yee A.J., Huff C.A., Moreau P., Dingli D., Cole C., Lonial S. (2019). Oral Selinexor-Dexamethasone for Triple-Class Refractory Multiple Myeloma. N. Engl. J. Med..

[185] Lapalombella R., Sun Q., Williams K., Tangeman L., Jha S., Zhong Y., Goettl V., Mahoney E., Berglund C., Gupta S. (2012). Selective inhibitors of nuclear export show that CRM1/XPO1 is a target in chronic lymphocytic leukemia. Blood.

[186] Abdul Razak A.R., Mau-Soerensen M., Gabrail N.Y., Gerecitano J.F., Shields A.F., Unger T.J., Saint-Martin J.R., Carlson R., Landesman Y., McCauley D. (2016). First-in-Class, First-in-Human Phase I Study of Selinexor, a Selective Inhibitor of Nuclear Export, in Patients With Advanced Solid Tumors. J. Clin. Oncol..

[187] Wu M., Gui H., Feng Z., Xu H., Li G., Li M., Chen T., Wu Y., Huang J., Bai Z. (2018). KPT-330, a potent and selective CRM1 inhibitor, exhibits anti-inflammation effects and protection against sepsis. Biochem. Biophys. Res. Commun..

[188] Sinniah A., Yazid S., Flower R.J. (2017). The Anti-allergic Cromones: Past, Present, and Future. Front. Pharmacol..

[189] Granucci E.J., Griciuc A., Mueller K.A., Mills A.N., Le H., Dios A.M., McGinty D., Pereira J., Elmaleh D., Berry J.D. (2019). Cromolyn sodium delays disease onset and is neuroprotective in the SOD1. Sci. Rep..

[190] Hori Y., Takeda S., Cho H., Wegmann S., Shoup T.M., Takahashi K., Irimia D., Elmaleh D.R., Hyman B.T., Hudry E. (2015). A Food and Drug Administration-approved asthma therapeutic agent impacts amyloid β in the brain in a transgenic model of Alzheimer disease. J. Biol. Chem..

[191] Ng G., Ohlsson A. (2017). Cromolyn sodium for the prevention of chronic lung disease in preterm infants. Cochrane Database Syst. Rev..

[192] Topalian S.L., Hodi F.S., Brahmer J.R., Gettinger S.N., Smith D.C., McDermott D.F., Powderly J.D., Carvajal R.D., Sosman J.A., Atkins M.B. (2012). Safety, activity, and immune correlates of anti-PD-1 antibody in cancer. N. Engl. J. Med..

[193] Yi M., Zheng X., Niu M., Zhu S., Ge H., Wu K. (2022). Combination strategies with PD-1/PD-L1 blockade: Current advances and future directions. Mol. Cancer.

[194] Ettinger D.S., Wood D.E., Aisner D.L., Akerley W., Bauman J.R., Bharat A., Bruno D.S., Chang J.Y., Chirieac L.R., D’Amico T.A. (2022). Non-Small Cell Lung Cancer, Version 3.2022, NCCN Clinical Practice Guidelines in Oncology. J. Natl. Compr. Cancer Netw..

[195] Keilholz U., Ascierto P.A., Dummer R., Robert C., Lorigan P., van Akkooi A., Arance A., Blank C.U., Chiarion Sileni V., Donia M. (2020). ESMO consensus conference recommendations on the management of metastatic melanoma: Under the auspices of the ESMO Guidelines Committee. Ann. Oncol..

[196] Audisio M., Tucci M., Di Stefano R.F., Parlagreco E., Ungaro A., Turco F., Audisio A., Di Prima L., Ortega C., Di Maio M. (2022). New emerging targets in advanced urothelial carcinoma: Is it the primetime for personalized medicine?. Crit. Rev. Oncol. Hematol..

[197] Herrmann M., Schulte S., Wildner N.H., Wittner M., Brehm T.T., Ramharter M., Woost R., Lohse A.W., Jacobs T., Schulze Zur Wiesch J. (2020). Analysis of Co-inhibitory Receptor Expression in COVID-19 Infection Compared to Acute. Front. Immunol..

[198] Curtin N., Bányai K., Thaventhiran J., Le Quesne J., Helyes Z., Bai P. (2020). Repositioning PARP inhibitors for SARS-CoV-2 infection(COVID-19); a new multi-pronged therapy for acute respiratory distress syndrome?. Br. J. Pharmacol..

[199] Heer C.D., Sanderson D.J., Voth L.S., Alhammad Y.M.O., Schmidt M.S., Trammell S.A.J., Perlman S., Cohen M.S., Fehr A.R., Brenner C. (2020). Coronavirus infection and PARP expression dysregulate the NAD metabolome: An actionable component of innate immunity. J. Biol. Chem..

